# Theta-Frequency Resonance at the Cerebellum Input Stage Improves Spike Timing on the Millisecond Time-Scale

**DOI:** 10.3389/fncir.2013.00064

**Published:** 2013-04-10

**Authors:** Daniela Gandolfi, Paola Lombardo, Jonathan Mapelli, Sergio Solinas, Egidio D’Angelo

**Affiliations:** ^1^Neurophysiology Unit, Department of Brain and Behavioral Sciences, University of PaviaPavia, Italy; ^2^Department of Biomedical, Metabolic and Neural Sciences, University of Modena and Reggio EmiliaModena, Italy; ^3^Brain Connectivity Center, IRCCS Fondazione Istituto Neurologico Nazionale C. MondinoPavia, Italy

**Keywords:** resonance, cerebellum, granular layer

## Abstract

The neuronal circuits of the brain are thought to use resonance and oscillations to improve communication over specific frequency bands (Llinas, 1988; Buzsaki, 2006). However, the properties and mechanism of these phenomena in brain circuits remain largely unknown. Here we show that, at the cerebellum input stage, the granular layer (GRL) generates its maximum response at 5–7 Hz both *in vivo* following tactile sensory stimulation of the whisker pad and in acute slices following mossy fiber bundle stimulation. The spatial analysis of GRL activity performed using voltage-sensitive dye (VSD) imaging revealed 5–7 Hz resonance covering large GRL areas. In single granule cells, resonance appeared as a reorganization of output spike bursts on the millisecond time-scale, such that the first spike occurred earlier and with higher temporal precision and the probability of spike generation increased. Resonance was independent from circuit inhibition, as it persisted with little variation in the presence of the GABA_A_ receptor blocker, gabazine. However, circuit inhibition reduced the resonance area more markedly at 7 Hz. Simulations with detailed computational models suggested that resonance depended on intrinsic granule cells ionic mechanisms: specifically, *K*_slow_ (M-like) and KA currents acted as *resonators* and the persistent Na current and NMDA current acted as *amplifiers*. This form of resonance may play an important role for enhancing coherent spike emission from the GRL when theta-frequency bursts are transmitted by the cerebral cortex and peripheral sensory structures during sensory-motor processing, cognition, and learning.

## Introduction

Brain activity is characterized by complex temporal patterns, which often take the form of coherent oscillations (Buzsaki, 2006). These are organized over defined frequency bands giving raise to the electroencephalographic rhythms. Important among these is the theta-band, which characterizes various functional states encompassing deep sleep, attentiveness, learning, and voluntary movement. In the latter case, the commands generated by the motor cortex are associated with increased power in the theta-band (around 6–9 Hz in humans), which is then transmitted to cortical and subcortical centers (Gross et al., 2005; Schnitzler et al., 2006, 2009). Interestingly, these same frequencies are exploited by the thalamo-cortical circuits to elaborate motor commands and estimate kinematic parameters in behaviors like whisking (Ahissar et al., 2000; Szwed et al., 2003; Kleinfeld et al., 2006; Zuo et al., 2011). But how do these signals interfere with the activity of the receiving networks? One main hypothesis is that the receiving network is tuned over the transmission frequency band through resonance, being therefore more efficiently engaged when the transmitted frequency pattern is recognized (Llinas, 1988). This resonance could emerge both from membrane mechanisms based on specific ionic channels and from the synaptic connectivity of the neuronal circuit involved.

The cerebellum shows theta-band activity correlated with that of the premotor and motor areas during bimanual voluntary tasks in humans (Gross et al., 2005; Schnitzler et al., 2006, 2009). Moreover,  single-neuron responses in the cerebellum are correlated with theta-frequency activity in the sensory-motor cortex of awake rats (Ros et al., 2009). Extracerebellar theta-frequency activity (Harish and Golomb, 2010) can be conveyed to the cerebellum through two main pathways. The inferior olive, which also shows self-sustained theta-band oscillations (Marshall and Lang, 2004), can generate theta-frequency spike bursts in climbing fibers (Mathy et al., 2009), and complex spikes in Purkinje cells (Welsh et al., 1995; Van Der Giessen et al., 2008). The granular layer (GRL) could also be entrained into theta-rhythms by extracerebellar activity (Pellerin and Lamarre, 1997; Hartmann and Bower, 1998; Ros et al., 2009).

It has been reported that the interneuron inhibitory network made by Golgi cells shows theta-band resonant properties based on interneuronal connectivity through gap-junctions (Dugue et al., 2009). Moreover, it was shown that neuronal elements of the GRL show intrinsic resonance in the theta-band, as both the granule and the Golgi cells respond maximally to input currents at around 6 Hz (D’Angelo et al., 2001; Solinas et al., 2007a,b). However, it remained unclear whether the GRL resonates to extracerebellar inputs and which mechanisms would be involved. Here we show that patterned sensory stimulation of the whisker pad causes GRL resonance at 5–7 Hz in the rat cerebellum GRL with marginal involvement of the inhibitory network. Resonance enhanced spike generation in granule cells raising time-precision on the millisecond time-scale. Theta-band resonance in the GRL could play an important role to tune the cerebellar circuit over one of the main frequency band of cerebro-cortical activity during generation of voluntary movement and other central functional states.

## Materials and Methods

Recordings were performed *in vitro* and *in vivo* and mathematical simulations were done using computational models. Experiments were performed using Wistar rats at postnatal day P20–P24 [internal breeding, Harlan (Indianapolis, IN, USA)] both in acute slices and *in vivo* under anesthesia. All experiments were conducted in accordance with international guidelines from the European Community Council Directive 86/609/EEC on the ethical use of animals.

### Stimulation patterns

In order to investigate the frequency – dependent properties of the GRL response, stimulus trials were repeated at frequencies between 1 and 10 Hz (1 Hz steps). Each trial was composed of 30 bursts and was repeated in a pseudo-random order (10 times *in vivo*, 20 times in both VSD recordings, whole-cell recordings and simulations) to prevent potential effects due to short or long-term adaptation processes and to reduce the effect of response variability. The stimuli *in vitro* consisted of mossy fiber bursts composed of 3 pulses at 300 Hz intra-burst frequency. The stimuli *in vivo* consisted of 30 ms air puffs, which are known to generate short high-frequency bursts of similar frequency in the mossy fibers (Chadderton et al., 2004; Rancz et al., 2007).

### Recordings in acute cerebellar slices

Patch-clamp and VSD imaging recordings were obtained from parasagittal cerebellar slices (D’Angelo et al., 1995; D’Errico et al., 2009). Briefly, the rats were deeply anesthetized with halothane (Sigma-Aldrich, Saint Louis, MO, USA) and decapitated. The cerebellum was removed, the vermis isolated and fixed on the vibroslicer stage (Dosaka, Kyoto, Japan) with cyano-acrylic glue. Acute 220 μm thick slices were cut in cold cutting solution containing (in mM): 130 K-gluconate, 15 KCl, 0.2 EGTA, 20 HEPES, and 10 glucose, pH adjusted at 7.4 with NaOH. Slices were incubated at 32°C in oxygenated extracellular Krebs solution containing (in mM): 120 NaCl, 2 KCl, 1.2 MgSO_4_, 26 NaHCO_3_, 1.2 KH_2_PO_4_, 2 CaCl_2_, 11 glucose (pH 7.4 when equilibrated with 95% O_2_ and 5% CO_2_), at least 1 h before recordings. In some experiments the solution was implemented with the GABA_A_ receptor blocker 20 μM gabazine (SR95531; Sigma-Aldrich), which was maintained throughout the recording session. Slices were transferred to the recording chamber on the stage of an upright microscope (Zeiss Axioskop F2S, Oberkochen, Germany) and perfused at 1.5 ml min^-1^ with oxygenated Krebs solution at 32°C with a feed-back Peltier device (TC-324B, Warner Instruments Corp., Hamden, CT, USA). Slices were immobilized with a nylon mesh attached to a platinum Ω-wire.

#### Patch-clamp recordings

Whole-cell current-clamp recordings were made in whole-cell patch-clamp configuration from granule cells as reported previously (D’Angelo et al., 1995; D’Errico et al., 2009). Recordings were obtained using Multiclamp 700B amplifier (Molecular Devices, Union City, CA, USA) (3-dB; cut-off frequency = 10 kHz). Recordings were subsequently digitized at 20 kHz using pClamp 9 (Molecular Devices) and a Digidata 1322A A/D converter (Molecular Devices). Patch pipettes were pulled from borosilicate glass capillaries (Hilgenberg, Malsfeld, Germany) and filled with the following solution (in mM): 126 K-gluconate, 4 NaCl, 15 glucose, 5 HEPES, 1 MgSO_4_ * 7 H_2_O, 0.1 BAPTA-free, 0.05 BAPTA-Ca^2+^, 3 ATP, 100 μm GTP; pH was adjusted to 7.2 with KOH. This solution maintained resting free-[Ca^2+^] at 100 nM and had a resistance of 7–10 MΩ before seal formation. The stability of patch-clamp recordings can be influenced by modifications of series resistance and neurotransmitter release. To ensure that series resistance remained stable during the recordings, passive cellular parameters were extracted in voltage-clamp by analyzing current relaxation induced by a 10 mV step from a holding potential of −70 mV. According to previous reports (D’Angelo et al., 1993, 1995; Silver et al., 1996), the transients were reliably fitted with a monoexponential function yielding membrane capacitance (*C*_m_) of 3.9 ± 0.2 pF (*n* = 18), membrane resistance (*R*_m_) of 2.2 ± 0.3 GΩ (*n* = 18) and series resistance (*R*_s_) of 18.0 ± 0.9 MΩ (*n* = 18). The 3-dB cell plus electrode cut-off frequency was *f*_VC_ = (2π*R*_sC__m_)^−1^ = 2.3 ± 0.1 kHz (*n* = 18) and did not significantly change during recordings attesting their stability. Mossy fibers were stimulated with a bipolar tungsten electrode (Clark Instruments, Pangbourne, UK) via a stimulus isolation unit and stimulation intensity (±4–8 V; 100 μs) was raised until the EPSPs generated spikes in 10–50% of the responses at 1 Hz from a membrane potential between −70 and −60 mV (mean 64.2 ± 2.7 *n* = 18). From a comparison with previous data (D’Angelo et al., 1995; Sola et al., 2004), between one and three mossy fibers were stimulated per granule cell.

#### VSD imaging

The procedures for VSD imaging were the same as reported in previous papers (Mapelli and D’Angelo, 2007; Mapelli et al., 2010a,b). The slices were incubated for 30 min in oxygenated Krebs solution added with 3% Di-4-ANEPPS stock solution mixed with 50% fetal Bovine Serum (Molecular Probes). The dye (Di-4-ANEPPS, Molecular Probes) was dissolved and stocked in Krebs with 50% ethanol (SIGMA) and 5% Cremophor EL (a Castor oil derivative, SIGMA). Perfusion of standard extracellular solution (2–3 ml/min) maintained at 32°C with a feed-back temperature controller (Thermostat HC2, Multi Channel Systems, Reutlingen, Germany) was performed during the recording session. In a series of experiments, the extracellular solution was implemented with the GABA_A_ receptor blocker, 20 μM gabazine (SR95531; Sigma-Aldrich), which was maintained throughout the recording session. Mossy fibers were stimulated with a bipolar tungsten electrode (Clark Instruments, Pangbourne, UK) via a stimulus isolation unit using an electronic stimulator (STG 1008, Multi channel systems). The recording chamber was installed on an upright epifluorescence microscope (BX51WI, Olympus, Europa Gmbh, Hamburg, Germany), equipped with a 20X (XLUM Plan FL 0.95 NA) (see Tominaga et al., 2000). The light generated by a halogen lamp (150W, MHF-G150LR, MORITEX Corp., Tokyo, Japan) was controlled by an electronic shutter (model0, Copal, Co., Tokyo, Japan) and then passed through an excitation filter (λ = 530 ±  10 nm), projected onto a dichroic mirror (λ = 565 nm), and reflected toward the objective lens to illuminate the specimen. Fluorescence generated by the tissue was transmitted through an absorption filter (λ > 590 nm) to the CCD camera (MICAM Ultima, Scimedia, Brainvision, Tokyo, Japan). The whole imaging system was connected through an I/O interface (Brainvision) to a PC controlling illumination, stimulation, and data acquisition. The final pixel size was 5 μm with 20× objective. Full-frame image acquisition was performed at 1 kHz. The signal-to-noise ratio was improved by averaging 30 consecutive sweeps at the stimulus repetition frequency of 0.2 Hz. Given maximal Δ*F/F*_0_ ≈ 1% and noise SEM ≈ ± 0.1% (*n* = 12 slices), the signal-to-noise (S/N) ratio was about 10 times ensuring a reliable measurement of peak response amplitude. Data were acquired and displayed by Brainvision software and signals were analyzed using routines written in MATLAB (Mathworks, Natick, USA). The analysis was performed by evaluating the maximum response of the VSD signal obtained at each tested frequency (1–10 Hz) normalized to the first peak response.

### Recordings *in vivo*

Extracellular field recordings were performed in the GRL of Crus-IIa in 20–24-days-old Wistar rats (internal breeding, Harlan). The rats were deeply anesthetized with urethane (1.4 mg/kg; Sigma-Aldrich; see Roggeri et al., 2008) dissolved in 0.9% NaCl and injected intraperitoneally. The heart rate (360–420/min) and respiratory rate (100–120/min) of each animal were constantly monitored and remained stable throughout the experiments. Animals were placed on a stereotaxic table (David Kopf Instruments, Tujunga, CA, USA) covered with a heating pad and the body temperature was maintained at 38 ± 0.5°C through a feed-back temperature controller (Fine Science Tools Inc.; Foster City, CA, USA). The exposure of the cerebellar surface was performed following previously reported surgical procedures (Bower and Woolston, 1983; Morissette and Bower, 1996; Lu et al., 2005). Briefly, a craniotomy was made on the right hemisphere (AP −13 mm ML 3 mm). The *dura mater* was carefully removed and extracellular Krebs solution was placed onto the cerebellar surface (Roggeri et al., 2008).

Local field potential (LFP) recordings from the GRL were obtained at the depth of 500 μm with glass borosilicate pipettes (Hilgenberg) filled with NaCl 2 M (0.5–1 MΩ). Insertion of the electrodes was at a 45° angle. Sensory stimulation was performed through a plastic pipette connected to a MPPI-2 Pressure Injector (Applied Scientific Instrumentation, Eugene, OR, USA) and positioned 2–3 mm from the whisker pad. The low-frequency stimulation protocol delivered 30 ms air puffs (40 psi) at 0.1 Hz frequency. Extracellular currents were recorded with Multiclamp 700 A amplifier (Molecular Devices). The signals were band-pass filtered between 100 Hz (high-pass) and 2 kHz (low-pass), digitized at 50 kHz using a Digidata 1322A interface, and stored on a PC using Clampex 9 (Molecular Devices). The same board and software were used to monitor and record body temperature and heart and respiratory rate and to generate stimulation pulses. The GABA_A_ receptor blocker, 20 μM gabazine (SR95531, Tocris Cookson), was dissolved in Krebs solution, placed in a microliter syringe (Hamilton, Bonadus, Switzerland) and injected 500 μm deep into Crus-IIa of the cerebellar cortex close to recording electrode. Extracellular signals were acquired and processed off-line with the Clampfit 9.2 software (Molecular Devices). The signal-to-noise ratio was improved by averaging 100 consecutive traces and by digital filtering (Gaussian low-pass filter 1 kHz). The LFPs consisted of two main waves, T and C. T was generated by rapid afferences through the trigeminal-cerebellar pathway,  C by delayed afferences passing through the thalamo-cortico-ponto-cerebellar pathway (Morissette and Bower, 1996). In order to achieve a precise assessment of the LFP amplitude, only T measures were considered for resonance analysis. LFP peak amplitudes were measured relatively to the preceding 10 ms baseline.

### Computational modeling

Computational modeling was performed using a reduced version of a large-scale cerebellar GRL network model (Solinas et al., 2010). The granule cell (D’Angelo et al., 2001) and Golgi cell (Solinas et al., 2007a) models contain an explicit representation of ionic conductance mechanisms, which have been extensively tested with respect to large sets of experimental data. In order to generate the several seconds of neuronal activity required for these simulation, the modeling strategy was to use a single granule cell and a single Golgi cell in different combinations. The number of Golgi cells impinging on the granule cell (0–4) was simply simulated by linearly scaling the synaptic weight (see inset to Figure 6). The simulations were performed in the Python environment using models written in NEURON and were run on a 144 processors cluster (SiComputer INTEL MFSYS25V2).

In brief, as in the previous network model (Solinas et al., 2010), the synaptic mechanisms included neurotransmission dynamics based on a vesicular release process generating short-term facilitation and depression. Release probability was derived from experimental works showing average values of 0.4 at the mossy fiber – granule cell synapse (Sola et al., 2004; D’Errico et al., 2009) and 0.4 at the Golgi cell – granule cell synapse (Mapelli et al., 2009). Both the excitatory and inhibitory synapse were endowed with spillover mechanisms allowing activation of slow synaptic responses (Sargent et al., 2005). For the sake of simplicity, the synapses of the same kind (either excitatory or inhibitory) were set with identical release probability and postsynaptic receptor density and properties. In order to mimic synaptic noise and generate jitter in spike timing, release at the mossy fiber – granule cell synapse was set in its stochastic version (Arleo et al., 2010) and background activity was generated in the mossy fibers activating the granule cell and the Golgi cell.

The simulations were run by activating a single granule cell in all its 20 different synaptic combinations formed by four mossy fibers and four Golgi cell axon fibers (including the case of no active Golgi cell synapses to simulate inhibition block) (cf. Figure 8A). The Golgi cell was connected to the same four mossy fibers impinging of the granule cell plus other four, for a total of eight mossy fibers. All the mossy fibers carried low-frequency (4 Hz) background activity. Then, in each simulation, 1–4 mossy fibers were activated by a 3 pulses – 300 Hz burst repeated at frequencies between 0.2 and 10 Hz (in 0.2 Hz steps) for 50 times (during the activation bursts, in these mossy fibers the background activity was interrupted). The data relative to membrane potential, membrane conductance, and current were stored and analyzed off-line using Matlab routines (Mathworks Inc.).

The synaptic combinations determining the excitatory/inhibitory (E/I) balance of granule cells (Table 1) were considered to give specific relative contributions to the ensemble GRL response. The weight of these contributions was derived from estimates obtained in *in vivo* LFP recordings (Roggeri et al., 2008; Solinas et al., 2010; Diwakar et al., 2011) and from computations of the probability of connection between mossy fibers, Golgi cells, and granule cells (Solinas et al., 2010). These values are reported in Table 1.

**Table 1:** **Cross-table of excitatory and inhibitory inputs to a granule cell**.

Exc/Inh (%)	GoC 0	GoC 1	GoC 2	GoC 3	GoC 4	Total
MF 4	0.43	2.16	3.97	3.62	1.66	11.84
MF 3	7.9	12.78	1.17	1.48	0.51	23.84
MF 2	0	2.42	24.84	15	7.22	49.48
MF 1	0	0.97	5.32	6.29	2.26	14.84
Total	8.33	18.33	35.3	26.39	11.65	100

*The values indicate the relative contribution of each synaptic combination (from Solinas et al., 2010; Diwakar et al., 2011)*.

### Resonance analysis

In each experimental series (either LFP, VSD, patch-clamp, or modeling), for each tested frequency, the first five responses in each trial were excluded in order to allow the responses to stabilize. When required, the remaining 25 responses were averaged over all the trial repetitions at the same frequency. Resonance plots from LFP and VSD data were directly obtained by the response amplitude at the different frequencies. The response amplitudes at each frequency *f_i_* were normalized with respect to the value measured at 1 Hz, *y*(*f_i_*) = (*y*(*f_i_*) − *y*(*1 Hz*))*/y*(*1 Hz*). Resonance plots for single-neuron responses (either whole-cell recordings or simulations) were obtained by analyzing the frequency-dependent changes in spike count, first spike delay, first spike standard deviation, and average maximum depolarization (*sc, sd, ssd, amd*). In this case we calculated the Resonance Index (RI). At all tested frequencies, *sc, sd, ssd, amd* were measured, normalized between the extreme values for each cell and rescaled between 0 and 1. Thus, RI in a cell approaches one when the number of emitted spikes and membrane average depolarization are maximum and the delay and time-dispersion of the first spike are minimum. Then the four RI parameters (*sc, sd, ssd, amd*), were summed yielding a compound RI ranging from 0 to 4 and representative of resonance in the given cell.

The resonance plots were usually well fitted by a double Lorentz equation *y*(*f* ) (Siebert, 1986) (OriginPro8.0, Microcal Inc.) of the form:

(1) $$y\left(f\;\right)=y_{0}+\underset{i=1,2}{\sum }\frac{2{\text{A}}_{\text{i}}}{\pi }\cdot \frac{\omega_{i}}{4{\left(\;f-f_{ci}\right)}^{2}+\omega_{i}^{2}}$$

Where, *y_0_* is a baseline level and, for each *i*th curve, *A_i_* is the underlying area, *f_ci_* is the peak frequency, and *ω_i_* is the width at *y*(*f_ci_*)*/2*. Lorentzian fittings allowed to find the resonance frequencies (*RF*_1,2_ = *f*_c1,2_) and to determine the relative weight of the two component of resonance plots with respect to their amplitudes *y*(RF_2_)/y(RF_1_). Lorentzian fittings allowed also to calculate *RA* = *y*(*f_c_*)/ω, which corresponds to the quality factor *Q* in resonance literature and characterizes a resonator’s bandwidth relative to its center frequency. The goodness of fit was assessed with χ^2^ statistics and by calculating the squared correlation factor, *R*^2^. In some cases, the identification of peaks did not require fittings so that RF and RA were determined directly from the raw data by using a peak-detection routine (when the two procedures were compared, the data obtained by peak-detection did not significantly differ from those obtained using Lorentzian fitting: e.g., see Figure 2B).

### Statistical analysis

Data are reported as mean ± SEM and compared for their statistical significance by paired Student’s *t-*test, unless stated otherwise (a difference was considered significant at *p* < 0.05).

## Results

### Resonant responses evoked in the cerebellar cortex *in vivo*

**Figure 1:**
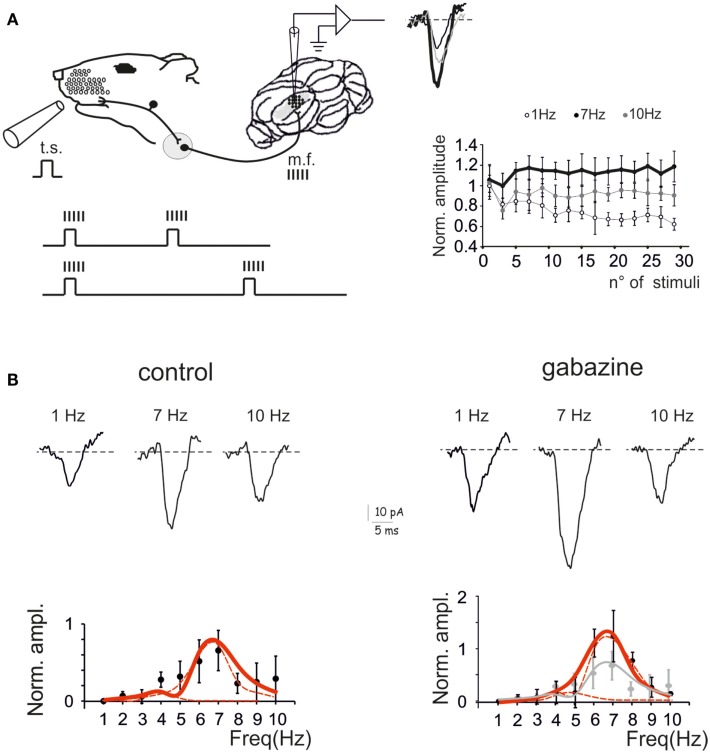
**Granular layer (GRL) resonance induced by sensory stimulation**. **(A)**. Schematic representation of the recording set-up *in vivo*. The lower inset illustrates that brief air-puff stimuli repeated at different frequencies are expected to generate repeated bursts in the mossy fibers (Chadderton et al., 2004; Rancz and Hausser, 2006; Rancz et al., 2007). By changing the frequency of sensory stimuli the frequency of the bursts is also changed. The GRL response is revealed by local field potential (LFP) recordings. The plot shows the time-course of LFP amplitude in response to subsequent pulses at three different stimulus frequencies. **(B)** LFPs recorded from the GRL at different frequencies of sensory stimulation in control and after intracerebellar microperfusion of gabazine. The normalized maximum LFP amplitude is reported in the plots. Note resonance at 5–7 Hz, which is maintained in the presence of gabazine. Data are reported as mean ± SEM. Red lines fitting the data point represent double Lorentzian functions (broken lines are the two individual component functions) obtained on control and gabazine data, respectively.

The response of the GRL to sensory inputs was evaluated by using repetitive air-puff stimulation of the whisker pad in urethane anesthetized rats. A single air-puff is known to cause a short spike burst in mossy fibers (Chadderton et al., 2004; Rancz et al., 2007) and to determine the LFPs recorded from the GRL (Roggeri et al., 2008; Diwakar et al., 2011) (Figure 1A). In order to investigate the frequency-dependence of LFPs in response to tactile stimulation, stimulus sequences of 30 impulses (30 ms duration) were delivered between 1 and 10 Hz in random order. After allowing responses to stabilize during the first 5 impulses, the amplitude of the average LFP obtained from the remaining 25 impulses was measured (Figure 1A). In control recordings, the LFP increased at 5–7 Hz and then decreased again toward 10 Hz. Thus, the response was resonant in the middle of the theta-band, with a peak at 7 Hz (65.6 ± 26.2% increase compared to 1 Hz; *p* < 0.01, *n* = 9) (Figure 1B, left).

The resonant properties of the GRL could reside into the intrinsic properties of granule cells (D’Angelo et al., 2001) but could also be shaped by the inhibitory Golgi interneuron network (Solinas et al., 2007b; Dugue et al., 2009). In order to test the role of the inhibitory circuit, the GABA_A_ receptor blocker, 20 μM gabazine, was microperfused into the GRL. After 5 min from commencing perfusion, the LFP increased by 49.4 ± 8.1% (*n* = 9; data obtained at 0.1 Hz, not shown), as expected from blockage of inhibitory transmission onto granule cells (Roggeri et al., 2008). Then, the stimulus sequence at different frequencies was repeated. The response was resonant in the middle of the theta-band, with a peak at 7 Hz (123.9 ± 4.9% increase compared to 1 Hz; *p* < 0.01, *n* = 9) (Figure 1B, right). Apparently, resonance increased with respect to control and became more pronounced at 7 Hz compared to 5 Hz.

Fittings to resonance plots were performed using double Lorentzian functions (see Eq. 1) under the hypothesis that two components were present in the data distributions and that their relative contribution could explain shape changes caused by gabazine application. In control, fittings yielded *RF*_1_ = 4.4 ± 0.9 and *RF*_2_ = 6.7 ± 0.3 with *y(RF*_2_/*y(RF*_1_) = 2.2 (*R*^2^ = 0.86; χ^2^ = 0.02). In the presence of gabazine, fittings yielded *RF*_1_ = 4.7 ± 1.6 and *RF*_2_ = 6.7 ± 0.3 with *y(RF*_2_)/*y(RF*_1_) = 4.8 (*R*^2^ = 0.9; χ^2^ = 0.003). Thus, gabazine application modulated the resonance profile by raising the relative weight of the component peaking around 7 Hz with respect to that peaking around 5 Hz.

### Resonant responses evoked in acute cerebellar slices

**Figure 2:**
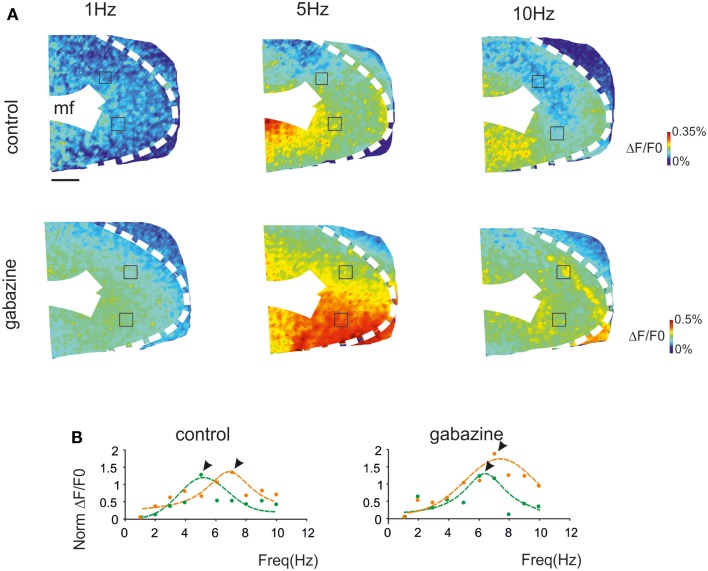
**The spatial distribution of resonance in the granular layer (GRL)**. **(A)** Optical maps of VSD responses in the GRL evoked by repeated bursts stimulation delivered to the mossy fibers bundle (mf) at three different frequencies. The VSD maps were generated by taking the maximum depolarization for each pixel both in control and after gabazine perfusion. Scale bar 100 μm. **(B)** The panels show the resonance curves corresponding to specific ROIs of the GRL indicated in **(A)** (squares). The data points show maximum responses either at 5 Hz (green) or 7 Hz (orange), both in control and after gabazine perfusion (arrowheads indicate points at the resonance frequency). Broken lines are single Lorentzian functions fitted to the points.

Voltage-sensitive dye (VSD) imaging was performed in parasagittal cerebellar slices in order to define the spatial distribution of resonance in the GRL. Mossy fiber stimulation was performed using 3 pulse-300 Hz bursts repeated at frequencies varying between 1 and 10 Hz in random order. The signals generated by mossy fiber stimulation invaded the GRL (Figure 2A) reproducing patterns reported previously (Mapelli et al., 2010a,b). In the same slices, 20 μM gabazine application increased the response to mossy fiber stimuli (average Δ*F*/*F*_0_ increase of 42.7 ± 11%; *p* < 0.01 *n* = 4), as expected from the disinhibitory effect of the drug (Mapelli et al., 2010a,b).

The GRL responses showed maximum Δ*F*/*F*_0_at input frequencies of 5–7 Hz (Figure 2A). This behavior was quantified by constructing resonance curves in ROIs covering limited areas of the GRL (or even single pixels), which showed characteristic profiles peaking in the same 5–7 Hz frequency range (Figure 2B). In the presence of gabazine, resonance at 5–7 Hz was still evident.

**Figure 3:**
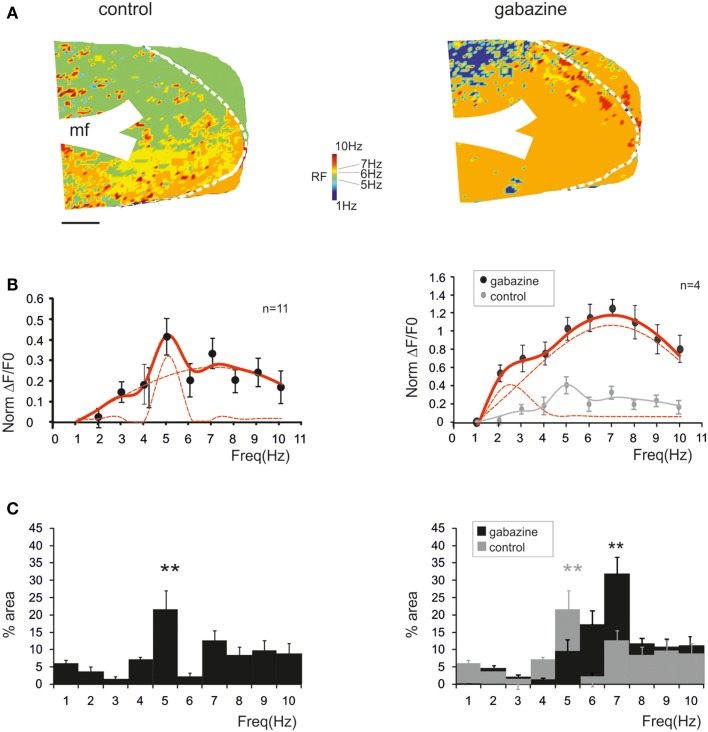
**Synaptic inhibition modulates granular layer (GRL) resonance**. **(A)** The maps show the resonance frequency (RF) for each of GRL pixels before and after application of gabazine. The maps reveal a prevalence of areas showing resonance at 5 and 7 Hz and an increase of the 7 Hz area after gabazine. Scale bar 100 μm. **(B)** The resonance profiles obtained by all active GRL pixels and averaged over several recordings (*n* = 11 control and *n* = 4 gabazine) reveal statistically significant peaks in the 5–7 Hz range. The amplitude of the resonance plot increases after gabazine. **(C)** Histograms report the relative areas covered by each RF averaged over several recordings (*n* = 11 control and *n* = 4 gabazine). Note that the main peak in control occurs at 5 Hz but moves to 7 Hz in the presence of gabazine. Data are reported as mean ± SEM. Red lines fitting the data point represent double Lorentzian functions (broken lines are the two individual component functions) obtained on control and gabazine data, respectively.

Resonance maps were constructed by plotting the resonance frequency (RF) measured in each pixel on color-scale (Figure 3A). The maps clearly showed that the large majority of single pixel resonance frequencies were in the 5–7 Hz region. After gabazine application, RF appeared still concentrated at 5–7 Hz, although the area covered by 7 Hz RF became prevalent.

The average pattern of changes was evaluated by generating average resonance curves, which were constructed from all single pixel resonance curves in each of 11 slices (Figure 3B). The average resonance curve showed two sub-peaks at 5 Hz (38.6 ± 9.3% increase vs. 1 Hz) and 7 Hz (31.1 ± 7.2% increase vs. 1 Hz), which were both significantly higher than nearest neighbor points (*p* < 0.05, paired *t*-test). In the presence of gabazine, the average resonance curve increased compared to controls (137.1 ± 9.6% increase; *p* > 0.4 *t*-test paired with nearest neighbors), with a maximum at 7 Hz (Figure 3B, right). In control, fittings yielded *RF*_1_ = 5.0 ± 0.6 and *RF*_2_ = 7.0 ± 0.6 with *y(RF*_2_)/*y(RF*_1_) = 0.7 (*R*^2^ = 0.92; χ^2^ = 0.04). In the presence of gabazine, fittings yielded *RF*_1_ = 4.4 ± 0.5 and *RF*_2_ = 7.0 ± 0.4 with *y(RF*_2_)/*y(RF*_1_) = 1.4 (*R*^2^ = 0.99; χ^2^ = 0.006). The ensemble changes caused by gabazine in the resonance curve were to increase the absolute size of the component peaking around 7 Hz and its relative weight with respect to that peaking around 5 Hz_,_ similar to what observed in the LFP *in vivo*.

The extension of 5–7 Hz resonance in the GRL was assessed by counting the number of pixels with the same resonant frequency (Figure 3C). On average, 5 Hz resonance occurred in 23.4 ± 4.7% of the active area and 7 Hz resonance occurred in 13.4 ± 2.9% of the active area. Overall, the only significant peak was that at 5 Hz (*p* < 0.01; paired *t*-test with nearest neighbors), suggesting that this was the most represented resonant frequency. In the presence of gabazine, the amount of area showing resonance at 7 Hz was increased compared to that at 5 Hz with a single significant peak at 7 Hz (31.7 ± 5.8% of active pixels; *p* < 0.01; paired *t*-test with nearest neighbors). In order to determine the origin of these differences, we measured resonance in individual pixels (evaluated using resonance amplitude, RA: see Materials and Methods), which showed similar amplitude in control and after gabazine perfusion (*p* < 0.00005, comparison of 50 pixels in control and after gabazine chosen at random). Therefore, since single pixel resonance was not significantly affected by gabazine (see above), the amplification of the resonance curve with gabazine was probably due to the increased area over which resonance occurred.

In aggregate, these results show that the cerebellar GRL in acute slices generates maximum responses when the input bursts conveyed through the mossy fibers are organized in theta-frequency range. The results closely resemble those obtained from LFPs *in vivo*, in that theta-frequency resonance persists but tends to increase at 7 Hz compared to 5 Hz after blocking inhibitory synaptic transmission.

### Resonant responses evoked in single granule cells by patterned synaptic activity

**Figure 4:**
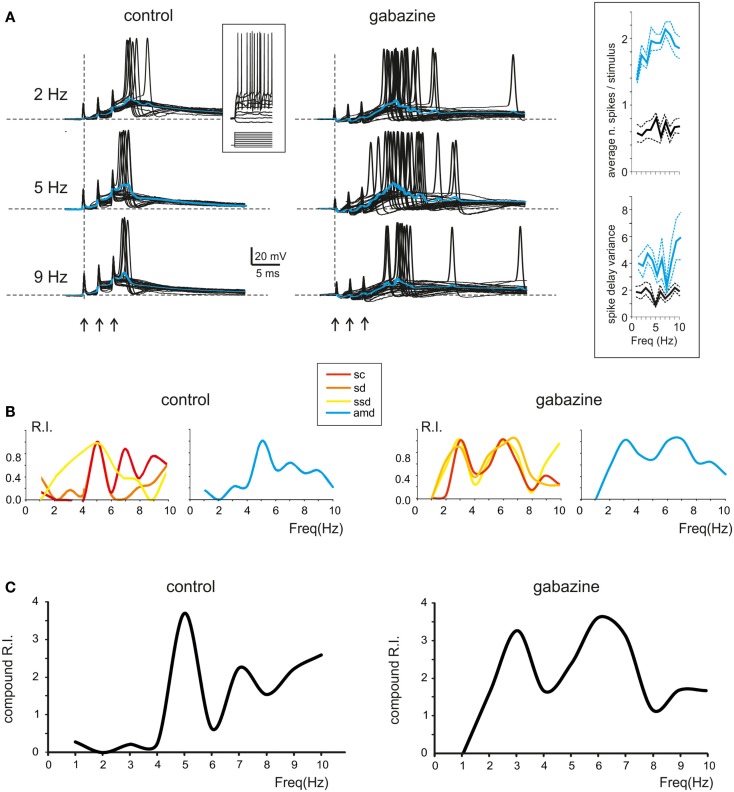
**Resonance in spike emission from single granule cells**. **(A)** *Left*; Single sweeps from a single granule cell recorded in control solution are shown over imposed at three different frequencies (2, 5, 9 Hz). Note the increased number, shorter delay and higher precision of spikes at 5 Hz. Blue traces show the average depolarization. Note that maximum depolarization is obtained at 5 Hz. *Right;* Single sweeps from a single granule cell recorded in the presence of gabazine are shown over imposed at three different frequencies (2, 5, 9 Hz). Note the increased number, shorter delay, and higher precision of spikes at 5 Hz. Blue traces show the average depolarization. Note that maximum depolarization is obtained at 5 Hz. The inset in the middle shows granule cell responses elicited by 150 ms-2pA current steps. The inset on the right shows the spike count and the spike variance in control and after gabazine perfusion. **(B)** *Left*; In a single granule cell, the Resonance Index (RI) for individual parameters in control (*sc, ssd, sd, amd*) reveal peaks between 5 and 7 Hz. *Right*; In the presence of gabazine RI reveal peaks between 5 and 7 Hz **(C)** Compound RI in the same granule cell in control (*left*) and in the presence of gabazine (*right*).

In order to investigate the cellular basis of resonance in the GRL, we performed whole-cell recordings from granule cells in acute cerebellar slices. The granule cells, which showed high input resistance (*R*_in_ = of 2.2 ± 0.3 GΩ *n* = 18) and low membrane capacitance (*C*_m_ = 3.9 ± 0.2 *p*F, *n* = 18), generated repeated spike discharge upon depolarizing current injection (D’Angelo et al., 1995, 2001) (Figure 4A, inset). The granule cells were maintained at a holding potential between −60 and −70 mV, from which mossy fiber low-frequency stimulation could elicit spikes in <50% of responses. Then, high-frequency burst patterns were repeated between 1 and 10 Hz in random order and the modifications in synaptic excitation were analyzed (Figure 4A; *left*).

In most recordings (*n* = 7), at 5–7 Hz, the burst depolarization increased along with the probability of generating spikes, which also became more precise and occurred with shorter delay (Figure 4A; *left*). In order to obtain an estimate of resonance from these measurements we calculated the RI (see Materials and Methods for definition) through the combination of the following parameters: spikes count (*sc*), first spike delay (*sd*), first spike SD (*ssd*), and average maximum depolarization (*amd*). It should be noted that these parameters are strongly correlated, so a stronger depolarization commonly elicited stronger spiking and shorter latency of the First spike (absolute values for *sc* and *ssd* are reported in Figure 4A, inset). Interestingly, RI/frequency plots for all the parameters (*sc, sd, ssd, amd*) showed a resonant shape and commonly peaked between 5 and 7 Hz (Figure 4B; *left*). The RIs were then summed to obtain a *compound RI* for each cell (Figure 4C, *left*). A similar analysis was performed in recordings in which gabazine was applied (*n* = 7). The granule cells became more excitable and made more spikes generating protracted discharges, but the resonance patterns remained unvaried. In most cases, the granule cells generated more spikes and the first spike occurred earlier and with higher precision around 5–7 Hz (Figure 4A; *right*) causing a higher maximum average depolarization at the same frequencies (Figure 4A; *right*). Accordingly, the RI/frequency plots showed main peaks at 5–7 Hz both for individual parameters (*sc, sd, ssd, amd*) (Figure 4B; *right*) and for the compound RI index (Figure 4C; *right*).

**Figure 5:**
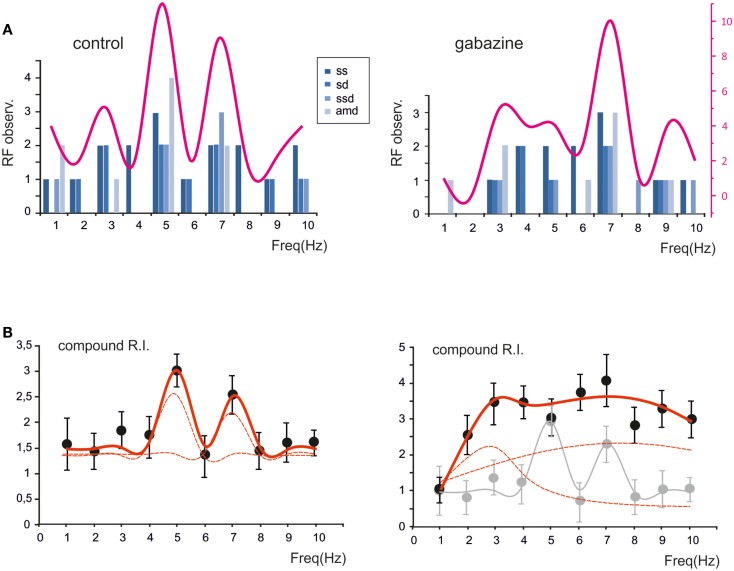
**The global effect of resonance on spike emission in the granular layer network**. **(A)** The histogram shows, for each frequency, the probability of observing a certain RF for spike number, spike delay, spike standard deviation and maximum average depolarization (*n* = 7). The superimposed plots show, at each frequency, the sum of observations in individual parameters. Note peaks at 5–7 Hz. **(B)** The plot shows the compound resonance index (RI) calculated from all the neurons recorded in control and in the presence of gabazine. Note peaks at 5–7 Hz. Data are reported as mean ± SEM. Red lines fitting the data point represent double Lorentzian functions (broken lines are the two individual component functions) obtained on control and gabazine data, respectively.

The probability of observing a given RF for each of the parameters (*sc, sd, ssd, amd*) is reported in Figure 5A, which shows that the maximum concentration of resonance frequencies occurs at 5–7 Hz, both in control and in the presence of gabazine. When the probability of occurrence were summed, a clear resonance curve peaking at 5–7 Hz was generated. Another way to represent the behavior of the granule cell population was to average their compound RIs. Also this average compound RI/frequency curve showed major peaks at 5–7 Hz (Figure 5B). In control, fittings to these plots yielded: *RF*_1_ = 4.8 ± 0.3 and *RF*_2_ = 7.0 ± 0.2 with *y(RF*_2_)/*y(RF*_1_) = 0.8 (*R*^2^ = 0.93; χ^2^ = 0.06). In the presence of gabazine, fittings yielded *RF*_1_ = 2.7 ± 0.5 and *RF*_2_ = 7.4 ± 0.6 with *y(RF*_2_)/*y(RF*_1_) = 1.1 (*R*^2^ = 0.86; χ^2^ = 0.1). Thus, the resonance profile of the ensemble responses obtained from several single granule cells showed that, after the application of gabazine, the relative weight of the component peaking around 7 Hz increased with respect to that peaking around 5 Hz.

In summary, the ensemble of spike-related parameters in granule cells generated resonance compatible with that observed in global network measurements obtained with LFPs (cf. Figure 1) and VSD imaging (cf. Figures 2–3), suggesting that resonance can be traced to elementary cellular phenomena mostly residing in the granule cells.

### Modeling granular layer resonance

**Figure 6:**
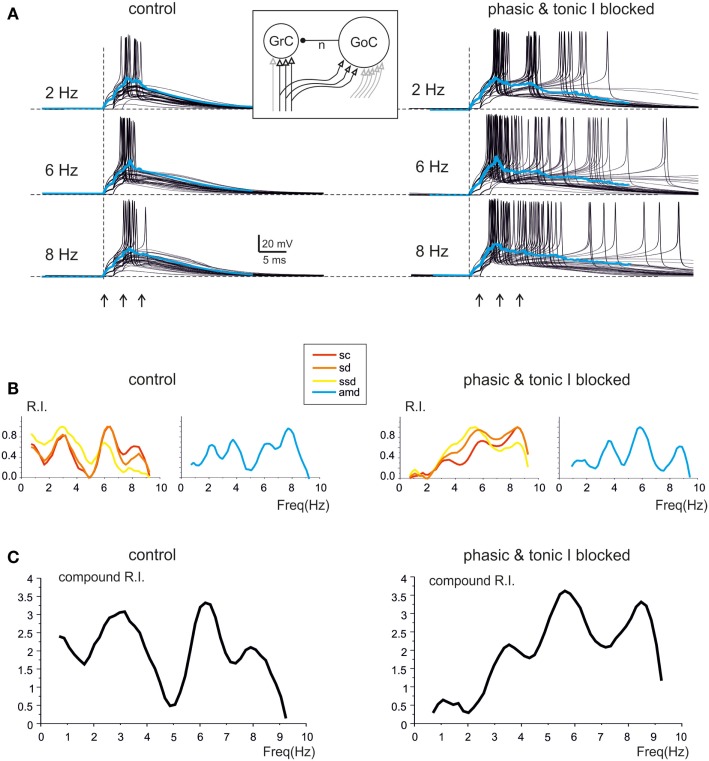
**Modeling resonance in spike emission from single granule cells**. Resonance in a model granule cell embedded into the granular layer network model. The traces and plots on the left show the case of two excitatory and two inhibitory synapses and simulate a control experimental condition. The traces and plots on the right have two excitatory synapses, while tonic and phasic inhibition are blocked simulating experimental gabazine application. The mossy fibers in the model are activated with patterns homologous to those used for investigating resonance in acute cerebellar slices (3-spike bursts at 300 Hz repeated 50 times at frequencies ranging from 1 to 10 Hz in 0.5 Hz increments). The inset shows a schematic of the model, with afferent fibers carrying background only (gray) or background interrupted by the bursts (black). **(A)** The last 45 of 50 sweeps from a single granule cell in the network model are shown superimposed at three different frequencies. Note the increased number, shorter delay and higher precision of spikes at 6 Hz (control and inhibition blocked) compared to the other two frequencies. The average traces are shown superimposed in color. **(B)** In the same model granule cell as in **(A)**, the R.I. for individual parameters (left: *sc, ssd, sd*; right: *amd*) reveal peaks in the theta range. **(C)** Compound R.I. obtained from the data shown in **(B)**. Both in **(A,B)** the peak around 7 Hz is enhanced after blocking synaptic inhibition.

The mechanisms underlying circuit resonance were explored by performing simulations with a model derived from a previous large-scale network of the GRL (Solinas et al., 2010). The model included detailed realistic representations of granule cells and Golgi cells and the related synapses (D’Angelo et al., 2001; Nieus et al., 2006; Solinas et al., 2007a,b; Arleo et al., 2010). Similar to what observed experimentally (D’Angelo et al., 1995; Sola et al., 2004), the mossy fiber – granule cell synapses were endowed with stochastic neurotransmission mechanisms, so that simulated granule cell responses were affected by noise and showed jitter in spike generation (Figure 6A). Once subjected to input patterns identical to those used in whole-cell recordings, the granule cells in the model generated RI/frequency plots for the resonance parameters (*sc, sd, ssd, amd*) and for compound RI, with main peaks in the 5–7 Hz region (Figure 6B), both in control and when simulating the “gabazine” condition (phasic and tonic inhibition blocked; Figure 6C).

**Figure 7:**
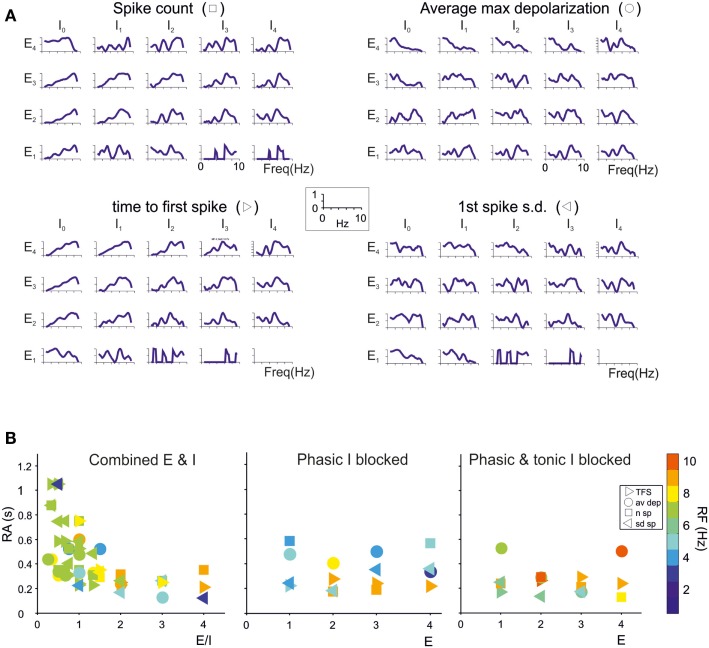
**Contribution of different E/I combinations to single-neuron resonance**. The impact of the excitatory/inhibitory balance on resonance is shown for all *E*/*I* combinations, indicated as *E*_n_/*I*_n_ (*n* is the number of synapses impinging on a given granule cell). Simulations were carried out as in Figure 7. **(A)** The effect of *E*/*I* combinations on RI for individual parameters (*sc, ssd, sd, amd*) are shown in the four panels. The columns *I*_0_ correspond to absence of phasic inhibition. Note that peaks in the plots more often occur between 5 and 7 Hz. **(B)** The plots reports RA (defined in Materials and Methods) for individual parameters (*sc, ssd, sd, amd*) in the cases of coexistence of *E* and *I* (left), of phasic inhibition blocked (middle) or of both phasic and tonic inhibition blocked (right). For each point, RF is reported using a color code. Note that 5–7 Hz RF clusters are at *E*/*I* ≤ 1 and correspond to the highest RA values, consistent with the enhancing effect of inhibition on resonance. Note also that, when both phasic and tonic inhibition are blocked, RF shifts toward higher values, as observed experimentally.

The granule cells in the model received all the basic combinations of excitatory and inhibitory connections occurring in the real network, amounting to just 20 different combinations (see Table 1). Indeed, an active granule cells receives 1–4 excitatory and 0–4 inhibitory synapses, on average (Solinas et al., 2010). The RI/frequency plots of resonance parameters (*sc, sd, ssd, amd*) for all the synaptic combinations are shown in Figure 7A. These plots reveal that, due to the different excitatory/inhibitory (E/I) balance, individual granule cells do not show identical resonant properties, although the RI/frequency plots usually peak in the 5–7 Hz range. Interestingly, resonance was more marked (evaluated using resonance amplitude, RA: see Materials and Methods) and more precisely centered at 5–7 Hz for E/I ≤ 1 (Figure 7B). Moreover, when synaptic inhibition was blocked (either in the transient or both in the transient and tonic component), RF shifted toward 7 Hz. These observations provide a possible explanation for the variability in resonance properties of single granule cells measured in whole-cell recordings and suggest that GRL resonance emerges from the statistical distribution of microscopic parameters characterizing granule cells with different E/I.

**Figure 8:**
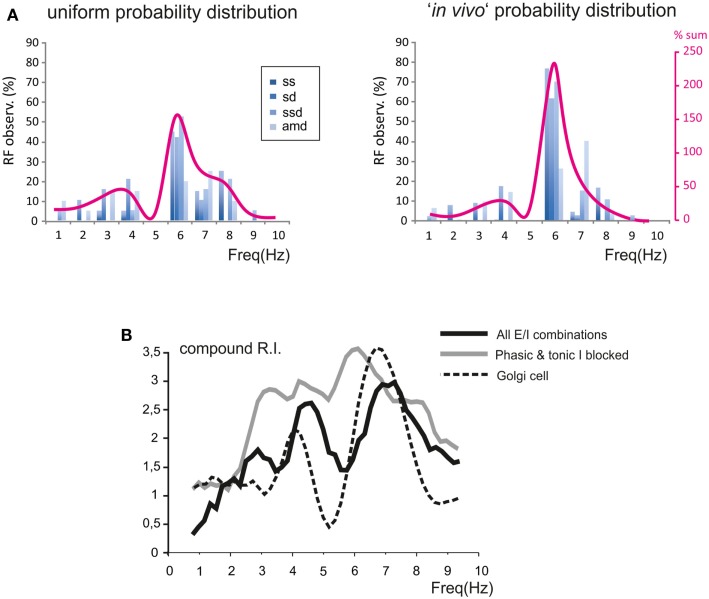
**Modeling the global effect of resonance on spike emission in the granular layer (GRL) network**. The global effect of resonance on spike emission in the GRL network has been modeled using different proportional contributions of granule cells with respect to their *E*/*I* balance. **(A)** The histograms show, for each frequency, the probability of observing a certain RF for individual parameters (*sc, ssd, sd, amd*). The superimposed plots show, at each frequency, the sum of observations in individual parameters. Note peaks at 5–7 Hz as in experimental recordings. In the histogram on the left we assumed a uniform probability distribution of *E*/*I* combinations. In the plot on the right we assumed the probability *E*/*I* distribution derived from Diwakar et al. (2011) *in vivo*. **(B)** The plot shows the compound RI calculated from model simulations using the *E*/*I* distribution derived from Diwakar et al. (2011) either including all E/I combinations (black trace) or with phasic and tonic inhibition blocked (gray trace; gabazine effect). Note the similarity of RI plots to those observed experimentally. The compound RI of a Golgi cell in the network (dashed trace) shows a major peak at 6–7 Hz, explaining why inhibition block enhances RI at the same frequencies.

In order to evaluate how the different E/I balance in granule cells influenced RF and RI/frequency plots, we determined the statistical distribution of these parameters (Figure 8A). The probability of observing a given RF for each of the resonance parameters (*sc, sd, ssd, amd*) is reported in Figure 8A for two different probability distributions. A uniform distribution, in which the probability of finding the different E/I balances is identical, was compared to the probability distribution reported by Diwakar et al. (2011) from the analysis of LFP recordings *in vivo* and VSD recordings in acute cerebellar slices. In both cases, the highest concentration of resonance parameters was observed at 5–7 Hz, with a higher peak using the Diwakar’s distribution.

The behavior of the whole granule cell population was reconstructed by summating and averaging the compound RIs for a given statistical distribution of E/I combinations. The RI/frequency curve showed major peaks at 5–7 Hz, and the 7 Hz peak became dominant after blocking synaptic inhibition (Figure 8B). The reason of the enhanced effect of inhibition at 7 Hz compared to 5 Hz was analyzed by applying RI analysis to Golgi cells in the simulated network. In response to mossy fiber bursts, Golgi cells had a main resonance peakat around 6–7 Hz, thereby depressing the granule cell response more effectively at this specific frequency (Figure 8B).

### Model predictions of the cellular mechanisms of resonance

**Figure 9:**
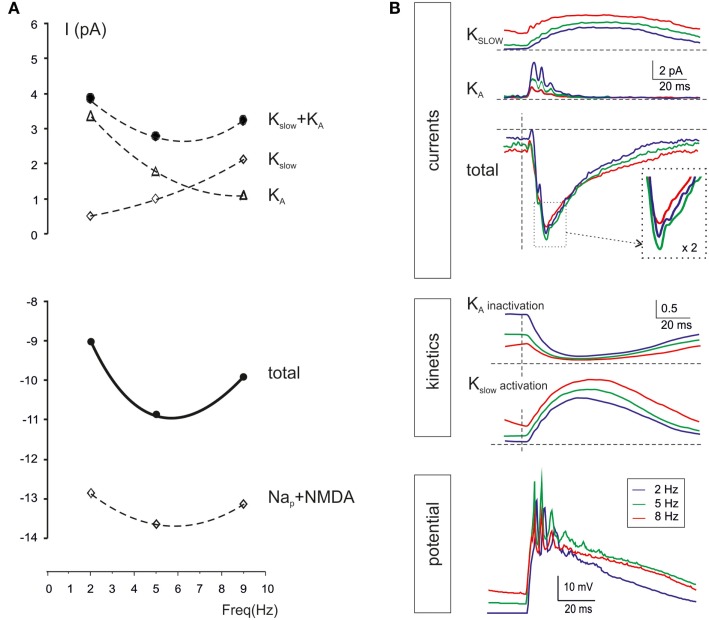
**Ionic mechanisms controlling single-neuron resonance**. The ionic mechanisms controlling single granule cell resonance in response to patterned synaptic activity were analyzed in simulations on the *E*_2_/*I*_1_ combination. **(A)** The values of currents generated by *K*_slow_, KA, Nap, and NMDA channels in response to simulated granule cells bursts were measured 20 ms after burst activation at three selected frequencies (2, 5, and 9 Hz). While *i*_KA_ decreases, *i K*_slow_ increases with frequency, so that *i*_KA_ + *i_K_slow* has a minimum at around 5 Hz. The *i*_Na_ and *i*_NMDA_ increase at 5 Hz, so that a net inward current of about 2 pA is generated at 5 Hz. **(B)** The plot on *top* shows *i*_KA_ and *i_K_slow* during simulated granule cells bursts delivered at frequencies of 2, 5, and 9 Hz. Note the enhancement of the total current at 5 Hz. The plots in the *middle* show the kinetics of *K*_slow_ activation and KA inactivation. Note that *K*_slow_ accumulated activation with frequency, whereas KA accumulates inactivation with frequency, explain the global resonant effect of their combination. The *bottom* tracings show membrane potential at the three different frequencies. All traces are taken from the simulations with two active excitatory and inhibitory synapses (average of 25 of 30 consecutive traces, the first 5 excluded).

Intrinsic granule cell resonance can be elicited by sinusoidal current injection depending on an *M*-like current (*K*_slow_) and and A-current (KA) acting as “resonators” and a persistent Na current acting as “amplifier” (Hutcheon and Yarom, 2000; D’Angelo et al., 2001). In the present simulations, during repetitive burst transmission, *K*_slow_ was higher at high-frequency while KA was higher at low-frequency, so that the two current-frequency curves intersected and generated a minimum *K*_slow_ + KA current at 5–7 Hz. At the same frequency, the persistent Na currents (Nap) and the NMDA current showed enhanced activation. The combined effect was that of generating a surplus of 2 pA in the 5–7 Hz range (Figure 9A). It should be noted that, over the high input resistance of granule cells (in the Giga-ohm range), a 2 pA current is indeed capable of causing a substantial enhancement in membrane depolarization and spike generation, as shown in previous studies (D’Angelo et al., 2001).

The time-course of *K*_slow_ and KA currents is shown in Figure 9B. While *K*_slow_ accumulated as the frequency of stimulation increased toward 10 Hz, KA decreased toward 10 Hz. The summed current was maximal at 5–7 Hz, just at the time of granule cell spike burst generation. The mechanism of frequency-dependence of *K*_slow_ and KA is analyzed in Figure 9B. *K*_slow_ activated slowly with time constants in the 100 ms range and therefore benefited of short inter-burst intervals. Conversely, KA inactivated rapidly with time constants in the 10 ms range and then took time to de-inactivate, so that KA benefited of long inter-burst intervals. The gating properties of *K*_slow_ and KA were therefore mechanistically correlated with generation of resonance in granule cells during mossy fiber burst transmission.

## Discussion

Following recognition of the role of oscillations and resonance as fundamental aspects of neuronal communication in the brain (Llinas, 1988; Buzsaki, 2006), their phenomenological properties and mechanisms have remained unclear in several circuits. This paper demonstrates that the cerebellum GRL, once activated with periodic inputs, shows resonance at 5–7 Hz. Resonance was manifest both *in vivo* following sensory stimulation with air puffs delivered to the whisker pad and in acute cerebellar slices following electrical stimulation with short bursts delivered to the mossy fiber bundle. Resonance was modified but persisted after blocking inhibitory synaptic transmission. Thus, theta-frequency resonance largely depended on intrinsic properties of the neurons involved (Hutcheon and Yarom, 2000). Interestingly, resonance was expressed by a change in the composition of spike bursts emitted by granule cells with a modulation of spike timing on the millisecond time-scale. Therefore, GRL resonance could recode with millisecond precision the theta-bursts at the input into new bursts at the output.

### General properties of granular layer resonance: The relationship with circuit inhibition

A striking property of GRL resonance evoked by theta-frequency patterns is that it has almost identical RF and gabazine sensitivity *in vivo* and *in vitro*. Resonance *in vivo* and *in vitro* are mechanistically correlated since each sensory stimulus generates a short high-frequency burst in mossy fibers (Chadderton et al., 2004; Rancz et al., 2007), so that the theta-frequency sensory volley is mimicked by theta-frequency stimulation of the mossy fiber bursts (Roggeri et al., 2008). This suggests that resonance observed *in vivo* is almost entirely generated in the GRL circuit rather than in the up-stream sensory pathway passing through the trigeminal nucleus (De Zeeuw et al., 1996). Also cerebro-cortical components were excluded, since the present analysis concerned only the trigeminal T wave of the cerebellar LFP (Morissette and Bower, 1996).

Both in LFPs *in vivo* and in VSD and whole-cell recordings in slices, the application of gabazine, a GABA-A receptor antagonist, to block inhibitory transmission between Golgi cells and granule cells did not suppress resonance but rather enhanced its component at 7 Hz. In general, GRL resonance was appropriately described by two components peaking at 5 and 7 Hz, and the 7 Hz peak prevailed when inhibitory transmission was blocked. In simulations, the Golgi cell showed maximum activity at 6–7 Hz, suggesting that the inhibitory circuit was indeed more effective at this particular frequency. It is thus possible that areas with 7 Hz resonance correspond to circuit regions that are less strongly inhibited than those with 5 Hz resonance. Double resonance peaks have been reported in the inferior olive, where the relative weight of the higher frequency peak is also depressed by synaptic inhibition (Llinas and Yarom, 1986). It could therefore be that in these circuits the inhibitory circuit has a modulatory role on resonance occurring along the main transmission pathway. Double resonance was also observed in cortical neurons, but in that case one peak was in the theta and one in the gamma band (Cobb et al., 1995). The inhibitory circuit passing through the cerebellar glomerulus also involves GABA-B receptors. Although a potential role of GABA-B receptors in GRL resonance remains unknown, it should be noted that GABA-B receptors down-regulate GABA-A receptor-mediated transmission at the Golgi cell – granule cell synapse [both by reducing GABA release (Mapelli et al., 2009) and postsynaptic receptor activation (Brandalise et al., 2012)] and enhance granule cell intrinsic excitability [by reducing an inward rectifier current (Rossi et al., 2006)]. At present, a major contribution of GABA-B receptors to resonance is therefore improbable.

### Granular layer resonance emerges from millisecond control of spike timing

Single cell analysis revealed that resonance in LFP and VSD recordings reflects the microscopic nature of spike generation in granule cells. At the RF, the granule cells showed higher probability of emitting spikes, which occurred with higher precision and shorter delay, resulting in a larger average depolarization. Since the LFP in the GRL is mostly due to extracellular spike currents (Diwakar et al., 2011), a higher probability of generating spikes and a higher synchrony of the spikes could well explain LFP resonance. As far as the VSD signal is concerned, since this technique is currently unable to precisely measure individual spikes, VSD resonance was more probably correlated with average cell depolarization.

### Prediction of resonance mechanisms using realistic computational modeling

The mechanisms of granule cell resonance during synaptic transmission were explored using a realistic computational models of the GRL circuit. The model, faithfully reproduced all macroscopic properties of resonance, including RF at 5–7 Hz and the shift toward 7 Hz caused by inhibition blockage. At the microscopic level, the model showed that resonance properties depended from the combination of single cell components in a statistically distribute manner and that the specific combination of excitatory and inhibitory synapses activating granule cells *in vivo* was especially effective in generating resonance in ensemble network activities. In the model, intrinsic granule cell resonance emerged from the interplay of *K*_slow_ and KA causing a minimum repolarizing current at 5–7 Hz. However, opposite to the case of sinusoidal current injection (D’Angelo et al., 2001), *K*_slow_ prevailed at higher frequencies while KA prevailed at lower frequencies. This happened because, different from sinusoidal currents, the bursts have fixed duration and what determines the frequency-dependence of channel activation and inactivation is the inter-burst interval. Thus, in subsequent bursts, *K*_slow_ (Hu et al., 2002) accumulated activation while KA accumulated inactivation. Granule cell resonance was amplified by the persistent Na current (Hu et al., 2002) and by voltage-dependent unblock of the NMDA current in the just-subthreshold region, which enhanced EPSP-spike coupling (D’Angelo et al., 1995; Mapelli and D’Angelo, 2007). As already pointed out (D’Angelo et al., 2001) It should be noted that, due to the high input resistance of granule cells, currents of just a few picoamperes in the threshold region resulted in significant effects on membrane depolarization and spike generation.

### Resonance and oscillations in the granular layer

Resonance is a condition occurring when a physical system undergoes a periodic activation with a frequency equal or close to the intrinsic oscillation frequency of the system itself, so that such a system tends to oscillate at its maximum amplitude. The nature of oscillations and resonance depends on the physical details of the system involved. So, what is the relationship between resonance and oscillations in the GRL? Theta-frequency oscillations are observed in the GRL during resting activity in the awake rat and monkey (Pellerin and Lamarre, 1997; Hartmann and Bower, 1998; Courtemanche et al., 2009). Computational analysis indicates that these oscillations require an intact feed-back inhibitory loop (Maex and De Schutter, 1998; Solinas et al., 2010). Conversely, as we report here, GRL resonance reflects intrinsic properties of granule cells. Finally, resonance and oscillations in the inhibitory interneuron network require gap-junctions between Golgi cells and other network components (Forti et al., 2006; Dugue et al., 2009) and possibly also intrinsic pacemaking in Golgi cells and other network components (Forti et al., 2006; Galliano et al., 2013). Thus, the circuit appears to be composed of two sub-systems, a resonator (the mossy fiber – granule cell synapse) coupled to a resonant oscillator (the Golgi cell inhibitory network). This latter also provides synchronicity through lateral inhibition and can enhance resonance in the granule cell population. In aggregate, resonance can amplify the granule cell output when the mossy fiber input is conveyed at 5–7 Hz. At this frequency the inhibitory circuit can spontaneously oscillate, thereby creating a condition at which the system can optimize phase locking and information transmission.

As pointed out above, by demonstrating similar resonance properties *in vivo* and *in vitro*, our results suggest that the major resonance mechanisms revealed by whisker pad stimulation reside in the GRL network. Indeed, the mossy fiber bursts evoked by eye-movements (Kase et al., 1980) correlate strictly with the sensory stimulus without any dependence on the phase or duration of the stimulus pattern. Nonetheless, the neuronal networks providing inputs to the GRL and even the whiskers might also present forms of resonance. For example, the thalamo-cortical circuit operates on a low-frequency bandwidth during whisking (Ahissar et al., 2000; Szwed et al., 2003; Kleinfeld et al., 2006) and both vibrissal motoneurons (Harish and Golomb, 2010) and trigeminal neurons (Wu et al., 2001) show forms of low-frequency oscillations and resonance. Therefore, low-frequency resonance in the cerebellar circuit is probably part of a resonant system distributed over the different nodes of the sensorimotor network controlling whisking. It should also be noted that mechanical resonance of vibrissae occurs at high-frequency and is probably relevant for detecting sharp changes in objects surface like edges or irregularities (Hartmann et al., 2003).

### Conclusions and functional implications

Granular layer network resonance, by reflecting millisecond regulation in spike emission, could fully exploit the outstanding timing capabilities of granule cells (Cathala et al., 2003; D’Angelo and De Zeeuw, 2009; Diwakar et al., 2009, 2011) and fine-tune information transfer (Arleo et al., 2010). One potential implication of GRL resonance is that, by occurring on the same band of Purkinje cell and inferior olive oscillations (Llinas and Yarom, 1986; Welsh et al., 1995; Lang et al., 2006; Abrams et al., 2012), could help maintaining a high level of coherence in the activity of the whole olivo-cerebellar system. On a larger scale, the cerebellum could be optimally designed to detect information carried by cerebro-cortical theta cycles implementing a well-tuned transmitter – receiver system (D’Angelo et al., 2009). For example, motor commands for whisking are emitted at theta-rhythm entraining the thalamo-cortical circuit (Szwed et al., 2003) and the cerebellum (O’Connor et al., 2002; Lang et al., 2006) providing the basis for complex resonant loops. Since oscillations in the GRL and in the Purinje cell – inferior olive circuits are tunable between 5 and 20 Hz (i.e., beyond the theta-band), the 5–7 Hz resonance reported here may be restricted to specific aspects of behavior. A hint comes from the specific sensitivity of the GRL to theta-bursts conveyed through sensory and cortical inputs, which can induce long-term synaptic plasticity in the cerebellum (Roggeri et al., 2008; Diwakar et al., 2011) as well as in the cortex and hippocampus (Larson and Lynch, 1988; Huerta and Lisman, 1995). An intriguing hypothesis is that resonance may improve learning of salient patterns in relation to voluntary movement (Gross et al., 2005; Schnitzler et al., 2006), attention and sleep, in which theta activity prevails.

## Author Contributions

D. Gandolfi and J. Mapelli performed the imaging recordings and data analysis, P. Lombardo performed electrophysiological recordings and data analysis, S. Solinas performed computational simulations, J. Mapelli and S. Solinas contributed to write the work, E. D’Angelo coordinated the work and wrote the final version of the manuscript.

## Conflict of Interest Statement

The authors declare that the research was conducted in the absence of any commercial or financial relationships that could be construed as a potential conflict of interest.
